# Poly(vinyl alcohol) Nanocomposites Reinforced with Bamboo Charcoal Nanoparticles: Mineralization Behavior and Characterization

**DOI:** 10.3390/ma8084895

**Published:** 2015-07-31

**Authors:** Cheng-Ming Tang, Yi-Hung Tian, Shan-Hui Hsu

**Affiliations:** 1 Institute of Oral Science, Chung Shan Medical University, Taichung 40201, Taiwan; E-Mails: ranger@csmu.edu.tw (C.-M.T.); bossisme7513@gmail.com (Y.-H.T.); 2 Institute of Polymer Science and Engineering, National Taiwan University, Taipei 10617, Taiwan; 3 Research and Development Center of Medical Devices, National Taiwan University, Taipei 10617, Taiwan

**Keywords:** Polyvinyl alcohol (PVA), bamboo charcoal nanoparticles (BCNPs), nanocomposite hydrogel, hydroxyapatite, biomineralization

## Abstract

Polyvinyl alcohol (PVA) demonstrates chemical stability and biocompatibility and is widely used in biomedical applications. The porous bamboo charcoal has excellent toxin absorptivity and has been used in blood purification. In this study, bamboo charcoal nanoparticles (BCNPs) were acquired with nano-grinding technology. The PVA and PVA/BCNP nanocomposite membranes were prepared and characterized by the tensile test, attenuated total reflectance-Fourier transform infrared spectroscopy (ATR-FTIR) and X-ray diffraction (XRD). Results showed that the tensile strength and elongation of the swollen PVA membranes containing 1% BCNPs (PB_1_) were significantly greater than those of PVA and other PVA/BCNP composite membranes. In addition, the major absorption band of OH stretching in the IR spectra shifted from 3262 cm^−1^ for PVA membrane containing 1% BCNP to 3244 cm^−1^ for PVA membrane containing 20% BCNP. This blue shift might be attributed to the interaction between the PVA molecules and BCNPs. Moreover, the intensity of the XRD peaks in PVA was decreased with the increased BCNP content. The bioactivity of the nanocomposites was evaluated by immersion in the simulated body fluid (SBF) for seven days. The mineral deposition on PB_5_ was significantly more than that on the other samples. The mineral was identified as hydroxyapatite (HA) by XRD. These data suggest that the bioactivity of the composite hydrogel membranes was associated with the surface distribution of hydrophilic/hydrophobic components. The PVA/BCNP composite hydrogels may have potential applications in alveolar bone regeneration.

## 1. Introduction

Hydrogels are three-dimensional polymer networks containing a significant amount of water. Hydrogels based on poly(ethylene glycol), poly(vinyl pyrrolidone), polyacrylamide and poly(hydroxyethyl methacrylate) are widely studied in the biomedical field [1,2]. In recent years, there has been increasing interest in the polyvinyl alcohol (PVA) hydrogels because of their good mechanical properties and adequate biocompatibility [3]. On the other hand, PVA is less integrated to living tissues because of its relatively limited biodegradability and bioactivity, compared to other polymers [4]. Efforts have been made to improve the bioactivity (e.g., osteoinductive potential) of the PVA hydrogels.

Adding particles is a common way to enhance the mechanical properties of a polymer [5], while the effect of nanoparticle additives is much more remarkable than that observed in conventional composites because of the high surface to volume ratio of the nanoparticles. For example, the thermal and mechanical properties of polyurethane were dramatically improved upon addition of a small amount (<100 ppm) of gold nanoparticles (≈5 nm) [6,7]. 

Bamboo charcoal is a renewable material that has a number of useful characteristics, such as high electric conductivity and self-lubricity, and can be used as an electromagnetic shield [8] and a friction material [9]. On the other hand, bamboo charcoal has a larger surface area (300 m^2^·g^−1^) than wood charcoal (30 m^2^·g^−1^) and thus has approximately four times more cavities and a four-fold higher absorption capacity [10]. These properties of bamboo charcoal make it a promising adsorbent material for enrichment and analysis of environmental pollutants [11]. The capability of bamboo charcoal for toxin adsorption has been reported in blood purification [12,13]. In addition, bamboo charcoal has uniform pore size (120–170 μm) and complete pore interconnectivity suitable for neobone ingrowth [14]. The bamboo charcoal particles emitted far infrared rays at a wavelength between 4 and 16 μm at 37 °C. Cancer cells such as HeLa cells treated with bamboo charcoal for six days showed a significantly lower multiplication rate compared with the control group [15]. Owing to the poor thermal resistance of cancer cells *vs.* primary cells, the bamboo charcoal powder was found a promising dental filler material for cancer prevention [16]. On the other hand, *in vitro* calcium phosphate deposition was found in the entire cross-section and vascular bundle of bamboo charcoal in simulated body fluid (SBF) after two weeks [17]. Calcium phosphate plays a key role in the osseointegration process and can induce osteoblastic cell attachment onto the material to form new mineralized layers. Another study indicated that bamboo may contain minerals such as silica, and after sodium hydroxide treatment, the surface showed hydrated silica globular particles, which may play an essential role in providing the sites for apatite nuclei [18]. The porous structure of bamboo is thus considered to induce the ingrowth of bone. On the other hand, carbon allotropy-graphene oxide (GO) is a two-dimensional nanomaterial prepared from the graphite. Solution of PVA/GO displayed pH-induced reversible sol-gel transition, *i.e.*, gel in the acidic environment and sol under alkaline conditions. Therefore, PVA nanocomposites may be applied at different physiological environments such as in gastric fluid or in buffered condition [19].

In this study, the nanosized bamboo charcoal particles (BCNPs) were obtained with the nano-grinding technology by the pulse gas flow. The nanocomposite hydrogels made from PVA containing different amounts of BCNPs (PVA/BCNP) were prepared and characterized by the field-emission scanning electron microscopy (FESEM) and attenuated total reflectance-Fourier transform infrared spectroscopy (ATR-FTIR). The tensile properties, swelling behavior, and free radical scavenging ability were measured. Finally, the nanocomposites were immersed in the simulated body fluid (SBF) and simulated gastric fluid (SGF) to evaluate the biological activity of PVA/BCNP nanocomposites under various physiological environments.

## 2. Results and Discussion

### 2.1. Characterization of BCNPs 

**Figure 1:**
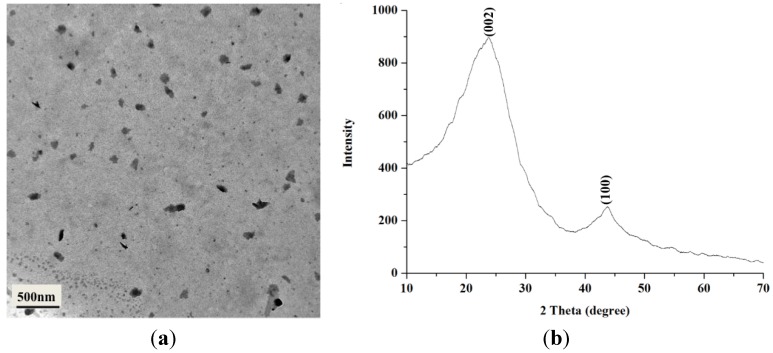
(**a**) A transmission electron microscopy (TEM) micrograph and (**b**) X-ray diffraction (XRD) pattern of bamboo charcoal nanoparticles (BCNPs).

Based on transmission electron microscopy (TEM) images, particles were in semicircular shape with an average diameter of BCNP 107.4 ± 9.8 nm (Figure 1a). In addition, the diffraction patterns of BCNPs are composed of three components, including a broad peak and two sharp peaks, similar to that of charcoal observed by Oberlin (Figure 1b) [20]. The three components in the X-ray diffraction (XRD) profile of charcoal were assigned to amorphous, graphite, and turbostratic components, respectively [20]. The (002) peaks at 2θ ≈ 23.7° correspond to parallel graphene layers. The (100) diffraction peaks at 2θ ≈ 43.7° characterize the 2D in-plane symmetry along the graphene layers [21]. According to the Bragg’s equation, the interlayer spacing *d*(002) can be used to estimate the degree of graphitization by the equation: *g* = [(0.3440 − *d*(002))/(0.3440 − 0.3354)] × 100, where g is the degree of graphitization (%), 0.3440 is the interlayer spacing of the fully non-graphitized carbon (nm), 0.3354 is the interlayer spacing of the ideal graphite crystallite, and the *d*(002) is the interlayer spacing derived from XRD (nm) [22]. According to the results of the calculation from the above formula, the degree of graphitization of BCNPs was 17.7%, indicating that graphite structure was present in BCNPs.

### 2.2. Characterization of PVA/BCNP Nanocomposite Membranes 

#### 2.2.1. Surface Morphology of PVA and PVA/BCNP Nanocomposite Membranes

**Figure 2:**
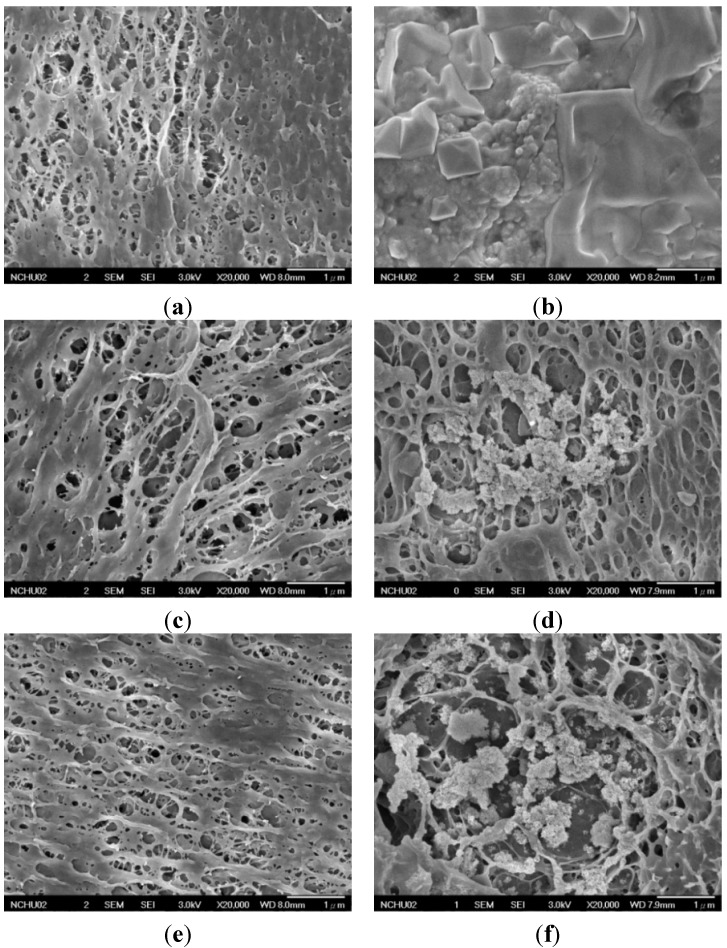

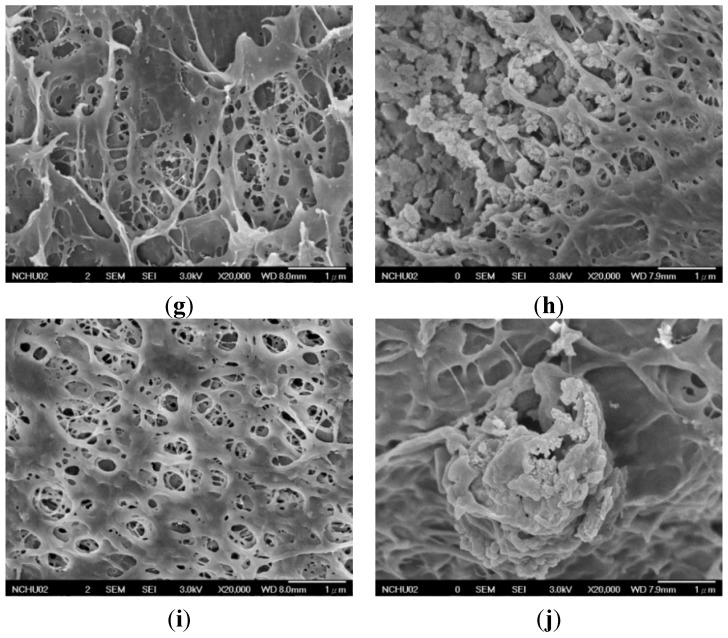
Surface morphology of (**a**,**b**) Polyvinyl alcohol (PVA) and PVA/BCNP nanocomposites of (**c**,**d**) PB_1_, (**e**,**f**) PB_5_, (**g**,**h**) PB_10_, and (**i**,**j**) PB_20_ before and after immersion in simulated body fluid (SBF) for seven days.

Nanocomposites with PVA/BCNP weight ratios of 99/1, 95/5, 90/10, and 80/20 were created and each were abbreviated as PB_1_, PB_5_, PB_10_, and PB_20_. Swollen PVA and PVA/BCNP nanocomposite membranes had smooth surface (data not shown). However, as the swollen membranes were dehydrated, an interconnected porous structure was observed for the membranes by FESEM (Figure 2). Commercial bamboo charcoal contains hydrophobic macro-particles that are subject to aggregation in an aqueous environment. This characteristic may make bamboo charcoal difficult to be uniformly dispersed in the PVA matrix.

#### 2.2.2. ATR-FTIR Analysis of PVA and PVA/BCNP Nanocomposites

**Figure 3:**
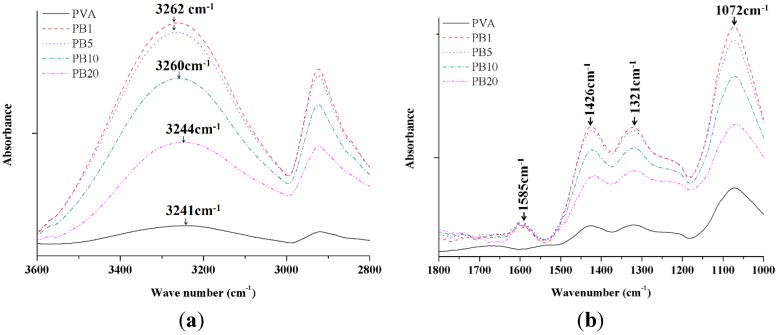
Attenuated total reflectance-fourier transform infrared spectroscopy (ATR-FTIR) spectra of PVA and PVA/BCNP nanocomposites. (**a**) At 3600–2800 cm^−1^ range and (**b**) at 1800–1000 cm^−1^ range.

The FTIR spectra provide some hint to the structural changes and possible interactions between PVA and BCNPs. The FTIR spectra of PVA and the PVA/BCNP nanocomposites membranes are shown in Figure 3. The absorption peaks of PVA included 3241 cm^−1^ (stretching of OH), 2935 cm^−1^ (symmetric stretching of CH_2_), 1426 cm^−1^ (bending of OH and wagging of CH_2_), 1321 cm^−1^ (stretching of C=O), and 1073 cm^−1^ (stretching of CO and bending of OH from amorphous sequence of PVA) [23]. Figure 3a demonstrates the characteristic absorption bands of PVA/BCNP nanocomposite membranes. It was apparent that after incorporation of BCNPs into PVA, the predominant broad absorption at 3241 cm^−1^ (=3086 nm) shifted to a higher wavenumber region in 3262 cm^−1^ (=3066 nm) in PB_5_. The wavelength decrease is considered a blue shift. This band was associated with the OH stretching of PVA (ν_OH_). Due to the high surface area of BCNPs, the surface OH groups may strongly interact with the macromolecular chains of PVA, forming interfacial layers on their surface and causing a blue shift. In Figure 3b, the characteristic absorption of bamboo charcoal occurred at 1588 cm^−1^, which was assigned to ring vibration in a large aromatic skeleton or stretching of carbon–carbon double bonds generally found in activated carbons such as graphite [24]. PVA/BCNP nanocomposites revealed the same absorption intensity at 1588 cm^−1^, which confirmed that a fixed amount of graphite existed on the nanocomposite surface. Besides, the bending of OH from the amorphous zone of PVA was observed at 1073 cm^−1^. This effect was the greatest for PB_1_. We suggested that when graphite existed on the nanocomposite surface, the amorphous PVA zone may also be exposed.

#### 2.2.3. XRD Analysis of PVA and PVA/BCNP Nanocomposites

**Figure 4:**
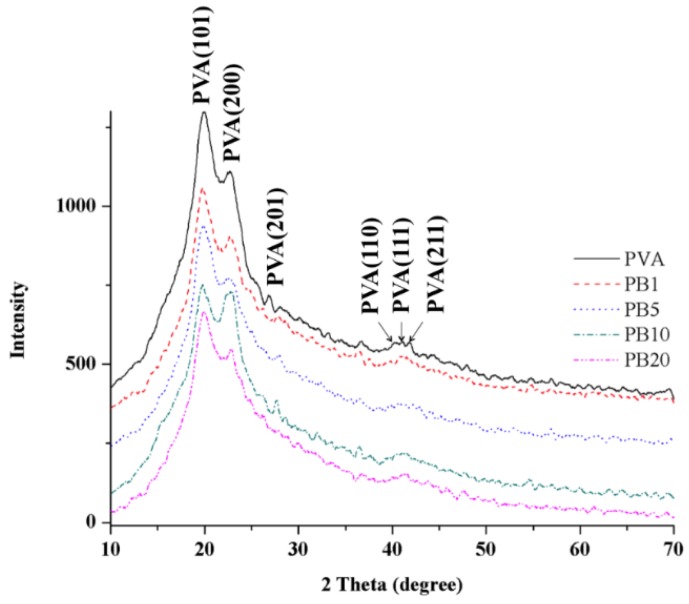
XRD patterns for PVA and PVA/BCNP nanocomposites.

The crystallinity is one of the major factors that affect the mechanical properties of a polymer. The XRD curves of the PVA and PVA/BCNP nanocomposites are presented in Figure 4. The XRD pattern of the pure PVA membranes revealed strong crystalline reflections at around 2θ = 19.92° and a shoulder at 22.74°. The two peaks are characteristic of PVA, representing reflections from (101) and (200) from a monoclinic unit cell [25]. In the XRD profile of PVA/BCNP nanocomposite membranes, the intensity of PVA diffraction peak decreased with the increased BCNP content. Moreover, no peak from bamboo charcoal was observed in XRD curves of the nanocomposites.

#### 2.2.4. Thermal Properties of PVA and PVA/BCNP Nanocomposite Membranes

Thermal stability of the PVA and PVA/BCNP composites was assessed by the thermogravimetric analysis (TGA) and differential scanning calorimeter (DSC) curves. The main data are summarized in Table 1. The pyrotytic temperatures was defined by TGA curve. The onset temperature of pyrolysis (*T*_onset_) first increased and then decreased with the increase in BCNP content. The highest *T*_onset_ was observed in PB_5_. On the other hand, the peak pyrolytic temperature (*T*_p_) kept increasing with the increased BCNP content. The highest *T*_p_ (338.5 °C) was observed in PB_20_. The crystallization temperature (*T*_c_), melting temperature (*T*_m_), and enthalpy of crystallization (∆*H*_c_) were defined by DSC. The change of *T*_c_ and *T*_m_ was somewhat random, with higher values observed in PB_1_ and PB_10_. 

The heat required to melt the sample was calculated by integration of the endothermic peak at *T*_m_. The degree of crystallinity (*X*_c_) of PVA was computed by comparison with the heat required to melt a 100% crystalline PVA sample by the following equation: *X*_c_ (%) = [∆*H*_c_/((1 − ϕ)·∆*H*_m_)] × 100, where ∆*H*_c_ is the apparent enthalpy of crystallization, ∆*H*_m_ is the extrapolated value of the enthalpy corresponding to the melting of a 100% crystalline sample (138.6 J/g) [26], and ϕ is the weight fraction of filler in the composite [27]. The computed *X*_c_ is also listed in Table 1. The value of *X*_c_ was the highest (42.7%) in PB_1_. The second highest *X*_c_ was observed in PB_10_ (10.8%).

**Table 1:** Thermal properties and crystallinity of PVA in PVA/BCNP nanocomposites.

Sample	Pyrolytic Temperature (°C)		*T*_c_ (°C)	*T*_m_ (°C)	∆*H*_c_ (J/g)	Crystallinity *X*_c_ (%)
	*T*_onset_	*T*_p_				
PVA	122.9	303.7	186.28	220.99	3.641	2.6
PB_1_	130.1	304.3	198.93	224.35	58.57	42.7
PB_5_	139.8	311.5	192.78	221.99	13.09	9.9
PB_10_	138.4	318.0	196.76	224.84	13.46	10.8
PB_20_	121.8	338.5	190.61	221.54	4.986	4.5

PVA: Polyvinyl alcohol; BCNP: bamboo charcoal nanoparticles; *T*_onset_: onset temperature of pyrolysis, obtained from thermogravimetric analysis (TGA) curves at 95% weight; *T*_p_: peak pyrolytic temperature, obtained from TGA curves at 50% weight. Crystallization temperature (*T*_c_), melting temperature (*T*_m_) and enthalpy of crystallization of PVA (∆*H*_c_), obtained from differential scanning calorimeter (DSC).

#### 2.2.5. Mechanical Properties of PVA and PVA/BCNP Nanocomposites

The mechanical properties such as tensile strength and elongation at break are important for biomaterials to function property. After PVA and PVA/BCNP nanocomposites were sufficiently swollen in deionized water at 37 °C for 24 h, the tensile properties were measured. Results of the tensile strength and elongation at break are shown in Table 2. The elongation at break and the tensile strength of nanocomposites were close for all samples. However, the values of PB_1_ membranes were significantly greater than those of the other groups. These data were consistent with the results of thermal analysis, where PB_1_ had the largest degree of crystallization. 

**Table 2:** Mechanical properties of PVA and PVA/BCNP nanocomposites by immersion in phosphate buffer solution (PBS) immersed for 24 h.

Sample	Tensile Strength (MPa)	Elongation at Break (%)
PVA	13.8 ± 0.9	305.0 ± 61.1
PB_1_	19.0 ± 0.9 *	369.0 ± 25.4
PB_5_	12.9 ± 1.8	336.9 ± 46.3
PB_10_	15.8 ± 1.3	305.0 ± 96.1
PB_20_	14.9 ± 1.8	303.4 ± 63.9

* Greater than all the other sample (*p* < 0.05).

#### 2.2.6. Swelling Properties of PVA and PVA/BCNP Nanocomposite Hydrogels 

**Figure 5:**
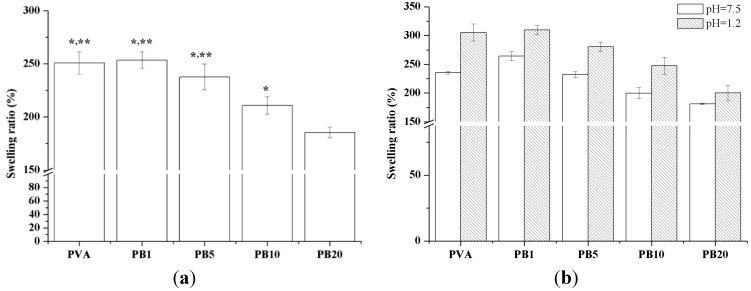
Swelling behaviors of PVA and PVA/BCNP nanocomposites in (**a**) deionized water and (**b**) solutions of various pH values including SGF (pH = 1.2) and SIF (pH = 7.5) at 37 °C. *: greater than PB_20_, **: greater than PB_10_ (*p* < 0.05).

Figure 5 shows the swelling ratios of PVA and PVA/BCNP nanocomposite membranes after immersion in buffer solutions of various pH value at 37 °C. When immersion in deionized water and simulated gastric fluid (SGF) (pH = 1.2), the swelling ratio was similar among PVA, PB_1_ and PB_5_. In literature, PVA hydrogels containing poly(acrylic acid) (PAA) or poly(methacrylic acid) (PMA) were used to develop pH sensitive formulations for drug delivery [28], and their drug release behavior was evaluated by immersion in simulated gastric fluid (SGF) and simulated intestinal fluid (SIF) to estimate possible drug distribution in human bodies. Figure 5 shows the swelling ratios of PVA and PVA/BCNP nanocomposite membranes after immersion in buffer solutions of various pH value at 37 °C. The most significant behavior of PVA and PVA/BCNP nanocomposite is their response against pH, exhibiting low swelling in neutral and basic conditions but high swelling in acidic conditions. When immersed in deionized water and SGF, the swelling ratios were similar among PVA, PB_1_ and PB_5_. Therefore, PVA/BCNP nanocomposites may have potential in controlled drug release for stomach treatment and may deserve further investigation.

#### 2.2.7. Free Radical Scavenging Effects of Nanocomposites

**Figure 6:**
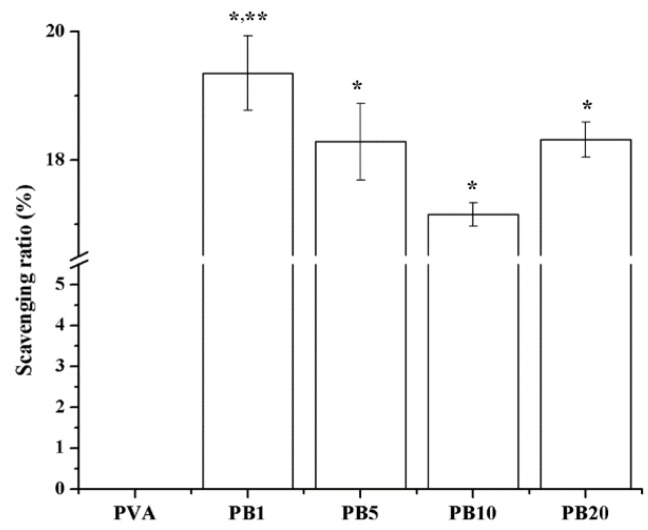
Free Scavenging ratios of PVA and PVA/BCNP nanocomposites. *: greater than PVA, **: greater than the other samples (*p* < 0.05).

Free radical scavenging ratios are demonstrated in Figure 6. The presence of BCNPs at all concentrations (1%–20%) in the PVA-BCNP nanocomposites enhanced the free radical scavenging ability of the PVA. Especially, the free radical scavenging ratio of PB_1_ was the greatest among all nanocomposites. This may be related to the dispersion states of BCNPs in the nanocomposites. Free radicals generated during the biological processes can lead to oxidative stress. Antioxidants play important roles in maintaining the normal function of biological systems by capturing harmful free radicals. In literature, the antioxidant properties of Au and Ag nanoparticles in waterborne polyurethane reduced the inflammation both *in vitro* and *in vivo* [6,29]. Therefore, free radical scavenging activities of the PVA/BCNP composites may lead to anti-inflammatory effects which further facilitate their biomaterial applications such as in wound treatment.

#### 2.2.8. Far Infrared Ray Emission Measurement of PVA/BCNP Nanocomposites

**Figure 7:**
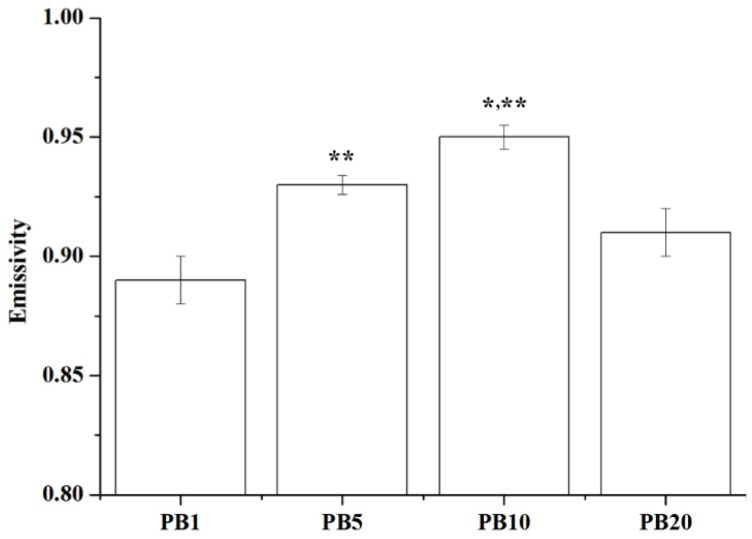
Far infrared ray emission measurement of PVA/BCNP nanocomposites. *: greater than PB_5_, **: greater than all the other sample (*p* < 0.05).

The wavelength of far infrared ray (FIR) is in the range of 4–1000 μm while the most human-effective wavelength is in the range of 4–14 μm [30]. The average emissivity of PVA/BCNP nanocomposites was measured by a far infrared thermo-emissivity apparatus. The emissivity (expressed by ε) is the ability of an object to emit infrared energy. As given by the Stefan-Boltzmann law, ε is the ratio of the thermal radiation from a surface to the radiation from the black body at the equal temperature. The ratio varies from 0–1. When ε is close to 1 (black body), the object fully converts heat into light. Results are shown in Figure 7. The average emissivities of the samples were greater than 0.85, supporting that PVA added with BCNPs indeed had the effect of FIR emission. The emissivity increased with the increase in the BCNP content and was especially high for PB_10_. This may be caused by the higher amount of BCNPs without severe aggregation. In a previous study, the proliferation of cancer cells such as Hela or fetal lung fibroblasts (WI-38) cells was inhibited by FIR [15]. Meanwhile, FIR also increased the healing speed of full-thickness skin wound in a rat model by the activation of fibroblasts and secretion of transforming growth factor beta 1 (TGF-β1) [31]. Since FIR may enhance the secretion of TGF-β1 from macrophages/platelets and result in TGF-β1 stimulated fibroblast migration, we hypothesized that PVA/BCNP nanocomposites may have potential in treating chronic wounds such as diabetic foot ulcers.

### 2.3. Biocompatibility of PVA and PVA/BCNP Nanocomposites

**Figure 8:**
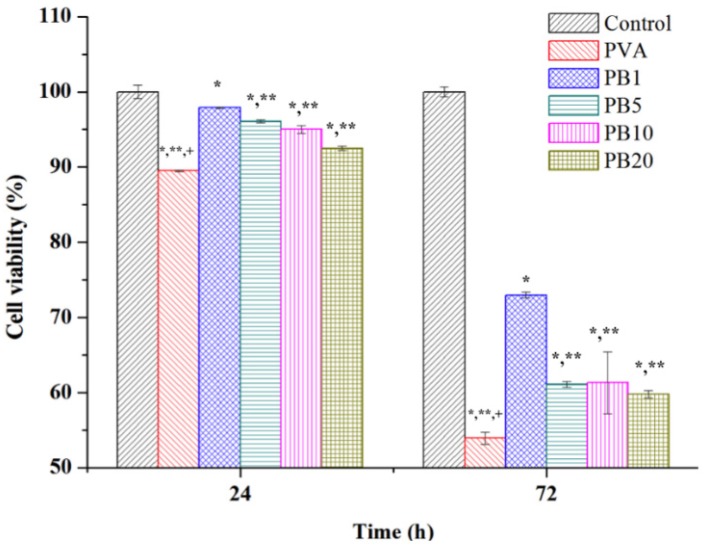
Biocompatibility of PVA and PVA/BCNP nanocomposites. *: smaller than control group (tissue culture polystyrene, TCPs); **: smaller than PB_1_, ^+^: smaller than all the other sample (*p* < 0.05).

To examine the biocompatibility of PVA and PVA/BCNP nanocomposites, the MTT assay was performed on L929 fibroblasts cultured with materials for 24 and 72 h. Results are shown in Figure 8. All samples demonstrated good cell viability. The viability on PVA/BCNP nanocomposites membranes was larger than that on the PVA membranes in both culture periods. 

### 2.4. Biomineralization of PVA and PVA/BCNP Nanocomposite Membranes

**Figure 9:**
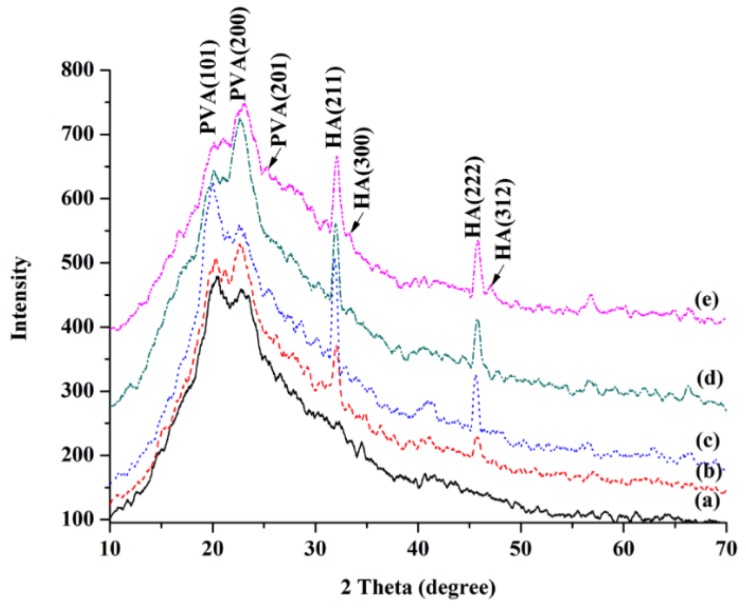
XRD patterns of (**a**) PVA and (**b**) PB_1_, (**c**) PB_5_, (**d**) PB_10_ and (**e**) PB_20_ nanocomposites by SBF immersion after seven days.

PVA and PVA/BCNP nanocomposites were immersed in simulated body fluid (SBF) for seven days. FESEM examination showed that the surface of all samples was covered by a mineralized layer, as shown in Figure 2. The XRD analysis was performed to confirm the mineralization of the PVA hydrogel and PVA/BCNP nanocomposites. Results from XRD analysis after immersion in SBF are presented in Figure 9. Before immersion in SBF, the XRD curve revealed a maximum intensity diffraction peak at 2θ = 19.9° corresponding to a d spacing of 0.44801 nm. This d spacing indicated the presence of a typical semi-crystalline structure. After immersion for seven days, PB_5_, PB_10_, and PB_20_ revealed diffraction peaks (2θ = 32.0°, 33.3°, 45.8° and 47.7°) representative of hydroxyapatite (Figure 9c–e). The crystallinity and the size of the crystal, however, were not easily obtained from the diffraction patterns.

**Figure 10:**
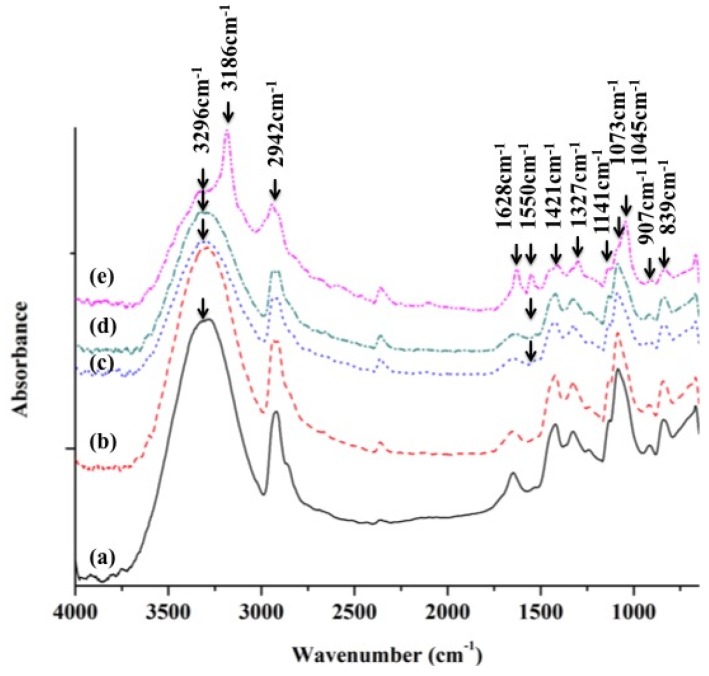
ATR-FTIR spectra of (**a**) PVA and (**b**) PB_1_, (**c**) PB_5_, (**d**) PB_10_ and (**e**) PB_20_ nanocomposites by SBF immersion after seven days.

The ATR-IR spectra of the mineralized PVA/BCNP membranes are shown in Figure 10. The new peak (1045 cm^−1^) [32], attributed to the presence of hydroxyapatite, was increased as the content of BCNPs increased. In all samples, a strong and wide hydroxyl band near 3296 cm^−1^ could be observed. In PB_20_, an additional salient hydroxyl band at 3186 cm^−1^ was observed. This band was probably associated with the –OH on the formed mineral or the interaction of –OH on PVA with the formed mineral [33]. The bands at 2942 cm^−1^ and 1628 cm^−1^ correspond to C–H asymmetric stretching and OH symmetric wagging vibrations. Meanwhile, the crystallinity of PVA was obtained from ATR-FTIR based on the peak at 1141 cm^−^^1^, which increases as the degree of crystallinity of PVA increases. The intensity of this peak is influenced by the crystalline portion of the polymeric chains. This peak is related to the symmetric C–C stretching mode or stretching of the C–O of a portion of the chain where an intramolecular hydrogen bond is formed between two neighboring –OH groups that are on the same side of the plane of the carbon chain [33]. While the peak at 1141 cm^−1^ indicates the stretching of C–O from the crystalline sequence of PVA, the peak at 1073 cm^−1^ indicated the stretching of C–O from the amorphous sequence of PVA [23]. Therefore, the ratio of the two peaks reflected the degree of crystallinity of PVA. A calculation of the ratios revealed that the degree of crystallinity on the surface of PVA/BCNP nanocomposite was increased after SBF immersion for seven days, especially in PB_20_. The table has been added in the Supplement (Figure S1 and Table S1).

Taken together, the above results (Figure 2, Figure 9 and Figure 10) suggested that PVA/BCNP nanocomposites may undergo mineralization in a biological environment. This was consistent with the earlier report that bamboo may provide the sites for apatite nuclei [18]. Because of the favorable biomineralization on PVA/BCNP nanocomposites, the potential of such nanocomposites to promote guided bone regeneration deserves further investigation.

## 3. Materials and Methods

### 3.1. Preparation of BCNPs 

Bamboo charcoal was acquired by the pyrolysis of moso bamboo (phyllostachys pubescens) in the absence of air. BCNPs were prepared by nano-grinding the bamboo charcoal with the technology by the pulse gas flow (Diamond Nano-Biochem Co., Taichung, Taiwan). The size and morphology of BCNPs were measured by TEM (JEM-1200, JEOL, Tokyo, Japan). 

### 3.2. Preparation of PVA and PVA/BCNP Nanocomposite Membranes

PVA used in this study was obtained from Sigma-Aldrich (St. Louis, MO, USA). The PVA has a molecular weight of 70,000–110,000 g/mol and hydrolysis grade of 98.5%. The homogeneous PVA solution was prepared by adding two grams of PVA in 10 mL of distilled water and stirring at 85 °C for 1 h. A known amount of BCNPs was then added to the PVA solution and stirred for 24 h at room temperature. The mixtures were poured onto to a polytetrafluroethylene (PTFE) mold (90 mm in diameter) and dried at room temperature overnight to allow the solvent to completely evaporate. 

### 3.3. Characterization of the Nanocomposites 

#### 3.3.1. Chemical Analysis

Fourier transform infrared (FTIR) spectroscopy was used to characterize the changes in specific functional groups. FTIR spectra were obtained by a Thermo Nicolet 380 spectrometer (Waltham, MA, USA) in the spectral range of 4000–650 cm^−1^ and a resolution of 2 cm^−1^, using the ATR technique.

#### 3.3.2. X-ray Diffraction

The X-ray diffraction (XRD) pattern was obtained by a diffractometer with graphite-monochromated Cu Kα radiation source at 30 kV and 15 mA (Miniflex II, Rigaku Corporation, Tokyo, Japan) in a scanning speed of 4°/min. The average size of individual crystallites was calculated from XRD data using the Scherrer’s formula *D* = 0.9λ/βcosθ, where *D* is the crystallite size in nm, λ is the radiation wavelength (0.154056 nm for Kα1 emission), β is the band-width at half-height, and θ is the diffraction peak angle [34].

#### 3.3.3. Surface Morphology 

Surface morphologies of PVA and PVA/BCNP nanocomposite membranes were examined by a field emission scanning electron microscope (FESEM) (JSM-6330F, JEOL, Tokyo, Japan). Samples were dried in vacuum at room temperature for 24 h and coated with gold before examination.

#### 3.3.4. Mechanical Properties of PVA and PVA/BCNP Composite Membranes

The tensile properties of membranes were measured by a universal testing machine (GF-AI-7000M, GO TECH, Taichung, Taiwan) using a low force load cell of 10 N capacity. Samples were immersed in deionized water for 24 h to achieve complete swelling. The thickness of each sample was measured by a micrometer with an accuracy of 1 μm. Testing samples were cut with a dumbbell-shaped die of length 40 mm and width 20 mm, and the measurement was performed at a crosshead speed of 10 mm/min. The tensile strength, strength at break and elongation at break were calculated based on the generated tensile stress-strain curve.

#### 3.3.5. Swelling Behaviors of PVA and PVA/BCNP Nanocomposite Membranes in Water and at various pH

To characterize the response of membranes to environments with different pH values, the pre-weighed dry samples were allowed to swell to equilibrium in deionized water and in solutions with various pH values including simulated gastric fluid (SGF, pH = 1.2), and simulated intestinal fluid (SIF, pH: 7.5) at 37 °C. SGF consisting of 3.2 mg/mL pepsin in 0.03 M NaCl and SIF consisting of 10 mg/mL of pancreatin in 0.05 M KH_2_PO_4_ were respectively prepared by following the United States pharmacopoeia (USP) [35]. It was observed that 24 h equilibration was enough to reach the equilibrium swelling of the samples. After the excessive water on the surface was removed with filter paper, the fully swollen samples were again weighed. The swelling ratio can be calculated as a function of time. Swelling ratio (%) = [(*W*_w_ − *W*_d_)/*W*_d_] × 100%, where *W*_d_ is the weight in the dry state of a sample and *W*_w_ is the weight in the swollen state of the sample.

#### 3.3.6. Free Radical Scavenging Effects of the PVA and PVA/BCNP Nanocomposites

Radical scavenging activities were assessed on the basis of the capacity of a sample to scavenge the stable 2,2-diphenyl-1-picrylhydrazyl (DPPH) free radical [36]. One milliliter of distilled water (control), or 1 mL of deionized water containing PVA or PVA/BCNP membranes (7.5 mm in diameter, 0.2 mm in thickness), was added to 3 mL of 32 μM DPPH free radical in methanol, vortexed, and left to stand for 90 min at room temperature. Absorbance of the reaction mixture was then measured at 515 nm using an ultraviolet–visible spectrophotometer (UV/VIS) (Helios Zeta, Thermo, Waltham, MA, USA). The radical scavenging effect was determined using the following equation: Scavenging ratio (%) = [1 − (absorbance of test sample/absorbance of control)] × 100%.

#### 3.3.7. Far Infrared Ray Emission Test of the PVA and PVA/BCNP Nanocomposites

AGEMA 500 infrared system (FLIR Systems, Wilsonville, OR, USA) was used for measuring the far infrared ray emissivity of PVA and PVA/BCNP nanocomposites. The heating temperature for each sample was controlled at 25 °C. 

### 3.4. Biocompatibility of the PVA and PVA/BCNP Nanocomposites

L929 mouse fibroblasts were used to determine the cytotoxicity of the PVA and PVA/BCNP nanocomposites. The cells were grown as monolayer cultures in 25T-flasks (Costar, Cambridge, MA, USA), sub-cultured three times a week at 37 °C, in an atmosphere of 5% CO_2_ in air and 100% relative humidity. The culture medium was Dulbecco’s Modified Eagle Medium (DMEM) (Sigma, St. Louis, MO, USA) supplemented with 10% (v/v) fetal bovine serum (FBS, Biochrom, Berlin, Germany) and 100 U/mL penicillin and 100 mg/mL streptomycin. When the cells reached the stage of confluence, they were harvested using 0.25% trypsin (Sigma, St. Louis, MO, USA), followed by addition of fresh culture medium to create a new single cell suspension for further inoculation. 

All materials were sterilized by 70% ethanol for 5 min, placed into the bottom of a 96-well tissue culture plate (Corning, Corning, NY, USA), and washed thoroughly by phosphate buffer solution (PBS) for 1 min. One milliliter of L929 cell suspension (containing 5 × 10^4^ cells) in culture medium was added to each well and cultured for up to 3 days. A blank well (tissue culture polystyrene, TCPS) was used as the control. After inoculation for 24 and 72 h, the methyl thiazol tetrazolium (MTT) assay [37] was performed to evaluate cell activity. Briefly, the culture medium was remove, and the cultures were washed with PBS twice. About 100 μL serum-free DMEM medium and 10 μL MTT solution (5 mg/mL in PBS) were added to each sample, followed by incubation at 37 °C for 4 h to allow MTT formazan formation. The medium and MTT were replaced by 100 μL of dimethyl sulfoxide to dissolve the formazan crystals. After 30 min, the optical density at 550 nm (OD550) was determined using a microplate reader (Stat Fax 2100, The Awareness Technology, Palm City, FL, USA). The ratios of OD550 from each parallel experimental group (*n* = 6) to the OD550 of control group were used to assess the cell activities.

### 3.5. Biomineralization of PVA/BCNP Nanocomposites

SBF was prepared with ionic concentrations nearly equal to those of human blood plasma (K^+^ 5.0, Na^+^ 142.0, Ca^2+^ 2.5, Mg^2+^ 1.5, Cl^−^ 147.8, HCO^3−^ 4.2, HPO_4_^2−^ 1.0, and SO_4_^2−^ 0.5 mM; pH 7.25), according to Kokubo’s procedures [38]. The PVA and PVA/BCNP nanocomposite membranes at a size of 10 mm × 10 mm were immersed in 10 mL of SBF solution in a plastic container, which was tightly closed and kept at 37 °C. The samples were retrieved after 7 days, rinsed with distilled water, and lyophilized by a freeze-drying device (FDU-1200, EYELA, Tokyo, Japan) for 24 h.

### 3.6. Statistical Analysis 

Multiple samples were collected in each measurement and expressed as mean ± standard deviation. The single factor analysis of variance (ANOVA) method was used to assess the statistical significance of the results. *p* values less than 0.05 were considered significant.

## 4. Conclusions

This study demonstrated that BCNPs may form nanocomposites with PVA. The swelling ratio was not significantly changed in low concentrations of BCNPs (1%–5%). The XRD analysis revealed that BCNPs may change the crystal orientation of PVA. In a wet state, BCNPs at 1% increased the tensile strength and elongation of PVA. BCNPs in PVA led to a blue shift of the hydroxyl stretching peak from 3241 to 3262 cm^−1^. The free radical scavenging ratio of PB_1_ was the greatest among all nanocomposites. This may be related to the dispersion states of BCNPs in the nanocomposites. Furthermore, PVA added with BCNPs indeed had the effect of far infrared ray emission. The emissivity increased with the increase in the BCNP content and was especially high for PB_10_. The biocompatibility of PVA and PVA/BCNP nanocomposites was examined by the MTT assay of cultured L929 fibroblasts. Results showed that all samples had good cell viability. After soaking in SBF for a week, PVA/BCNP nanocomposite hydrogel membranes containing BCNPs greater than 1% showed mineral deposition, of which the structure was determined to be hydroxyapatite by the XRD analysis. The BCNP-dose dependent increase and decrease in the thermal stability of PVA-BCNP nanocomposites suggested that the interaction of PVA and BCNPs may constantly change by varying the amount of BCNPs in the nanocomposites. Future work can be undertaken to explore the attachment, growth, and differentiation of bony cells for applications in tissue engineering and guided tissue regeneration. 

## Supplementary Materials

Supplementary materials can be accessed at: http://www.mdpi.com/1996-1944/8/8/4895/s1.
