# Supplementary material for: Poly(vinyl alcohol) Nanocomposites Reinforced with Bamboo Charcoal Nanoparticles: Mineralization Behavior and Characterization
Source: Materials (Basel). 2015 Jul 31;8(8):4895–911. doi: 10.3390/ma8084895 (PMC5455495; doi:10.3390/ma8084895)
Supplement: Supplementary file 1 [file materials-08-04895-s001.pdf]

Supplementary Materials

Table S1. The relative intensities of 1141 cm^{-1} and 1073 cm^{-1} peaks in the attenuated total reflectance-Fourier transform infrared spectroscopy (ATR-FTIR) spectra of Polyvinyl alcohol/bamboo charcoal nanoparticle (PVA/BCNP) nanocomposites after simulated body fluid (SBF) immersion for 7 days.

Sample	Absorbance at 1141 cm^{-1}	Absorbance at 1073 cm^{-1}	Absorbance Ratio (1141 cm^{-1}/1073 cm^{-1})	Surface Crystallinity of PVA (%)
PVA	0.10217	0.23408	0.436	30.4
PB_1	0.10618	0.22600	0.470	32.0
PB_5	0.07024	0.15056	0.467	31.8
PB_{10}	0.07252	0.15512	0.468	31.9
PB_{20}	0.05046	0.10276	0.491	32.9

Surface crystallinity of PVA (%) = [Absorbance at 1141 cm^{-1}/(Absorbance at 1141 cm^{-1} + Absorbance at 1173 cm^{-1})] × 100%.

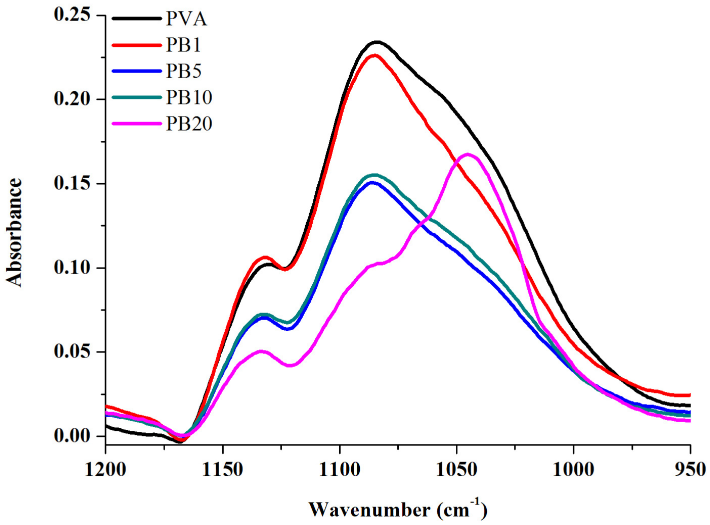

Figure S1. Enlarged ATR-FTIR spectra in the range of 950 to 1200 cm^{-1} for PVA/BCNP nanocomposites after SBF immersion for 7 days.
